# Correction: Propofol suppresses hormones levels more obviously than sevoflurane in pediatric patients with craniopharyngioma: A prospective randomized controlled clinical trial

**DOI:** 10.1371/journal.pone.0315226

**Published:** 2024-12-04

**Authors:** The PLOS ONE Staff

The first two authors, Jun Xiong and Mengrui Wang, should be noted as contributing equally to this work.
