# Supplementary material for: Propofol suppresses hormones levels more obviously than sevoflurane in pediatric patients with craniopharyngioma: A prospective randomized controlled clinical trial
Source: PLoS One. 2023 Jul 28;18(7):e0288863. doi: 10.1371/journal.pone.0288863 (PMC10381029; doi:10.1371/journal.pone.0288863)
Supplement: S1 File — (DOC) [file pone.0288863.s002.doc]

**Clinical trial protocol**

**Project summary:**

The most apparent defect of pediatric patients with craniopharyngioma (CP) is endocrine disorder. General anesthesia (GA) does impact on perioperative hormones levels. Because there is shortage of research related to interruption of GA on hormones levels in CP children, thus the study compared the effects of different anesthetics, sevoflurane and propofl, on perioperative hormones levels in pediatric patients with CP. CP children with ASA I-II undergoing CP resection under GA were enrolled from January to May 2022. Before recruitment, the study was registered on [www.chictr.org.cn](http://www.chictr.org.cn/) and got the approval from Ethic Committee. Their hormones levels were measured on preoperation, 1h after onset of operation, the end of operation and 72h after operation. Meanwhile, their vital life signs and postoperative complications were recorded too. Whether would sevoflurane and propofol interrupt hormones levels? And whose interruption would be more intensive? The result of the study is useful to select the suitable anesthetics for CP children undergoing resection.

**Title:**

Propofol suppresses hormones levels more obviously than sevoflurane in pediatric patients with craniopharyngioma: A prospective randomized controlled clinical study

**Name and address of the sponsor/funder:**

Wu Jieping Medical Foundation of Special Funding Support, No.320.6750.18502. No. 24 Jianwai Street, Chaoyang District, Beijing, China. 100022.

**Information of investigators:**

Jun Xiong, Xueyuan AVE 1098, Nanshan District, Shenzhen, Guangdong, China, 518055. 086-13810197854.

Yongxing Sun. No. 50 Yikesong, Xiangshan, Haidian District, Beijing, China, 100093. 086-13521697866.

Both are responsible for the study.

**Information of institution:**

Sanbo Brain Hospital, Capital Medical University.

**Rationale and background information:**

CP is histologically benign neuroepithelial tumor proximity to critical neurovascular structure, which is most common in children between the ages of 5 and 10 years accompanied with endocrine deficiencies, visual impairment and other hormonal symptoms. The majority of patients with CP present with at least one endocrine deficit. These endocrine deficiencies are also frequently caused by treatment related lesions to the hypothalamic pituitary axis. Consequently avoiding irreversible damage of vital neural structures is the key goal in the treatment of CP.

It has been well-known anesthesia protects patients from the stress response caused by surgical trauma with suppressing the endocrine stress reaction and sympathoadrenal system. And general anesthesia also affects hypothalamic pituitary thyroid axis to reduce the serum levels of TSH, free triiodothyromine (FT3) and total triiodothyronine (TT3). Whether is the inhibition of GA advantageous for pediatric patients with CP?

An important concern for anesthesiologists caring for children who have undergone resection of CP is how to minimize interrupting hypothalamic pituitary axis or the production and secretion of body natural pituitary derived hormones. Choosing a particular type of anesthetic in order to produce a preferred pattern of reaction of the endocrine system would be optimally homeostatic and befitting to these pediatric patients with CP. Unfortunately, there is shortage of studies to compare the effects between different anesthesia methods on perioperative levels of hormones, and the issue remains somewhat of a clinical dilemma. Thus we sought to undertake a study to determine the discriminations of inhalation anesthesia and total intravenous anesthesia impact on the levels of hormones in children with CP. This study would help to assure a high standard of treatment quality.

**Study goals and objectives:**

The aim of this study is to compare the different effects of sevoflurane and propofol on hormones levels in pediatric patients with CP undergoing elective CP resection under general anesthesia.

**Study design:**

This is a prospective randomized controlled clinical study. The study recruited pediatric patients with CP undergoing elective resection under general anesthesia.

The Inclusion Criteria: American Society of Anesthesiologists (I-II) elective CP resection with general anesthesia, age less than 18 years, and completion of preoperative measurement of hormones.

The Exclusion Criteria: patients or their guardians do not accept or cooperate with the study, suffering from other diseases which lead to hormone abnormity, intraoperative accidences possibly interrupting levels of hormones, for example, intraoperative massive hemorrhage.

The study was started after the completion of clinical trial registration and ethic approval. The scheduled duration of this study was from January to May 2022. By the calculation of sample size after pilot study, the expected number of patients was 64. All guardians of children wrote the informed written consent.

**Methodology:**

The pediatric patients were divided into two groups with 1:1 ratio via a SPSS random number generator, the sevoflurane inhalation anesthesia group (S group) and propofol total intravenous anesthesia group (P group). The allocation was done by an anesthesiologist without further assignment in this study. The group allocation was concealed with sequentially-numbered sealed envelops and blind to both the patients and investigators who were responsible for analyzing patients’ data.

Pediatric patients’ sex, age, weight, size were measured and recorded by the same healthcare team. Their body mass index (BMI) was calculated by monitor (GE CARESCAPE Monitor B650, Helsinki, Finland) based on their demographic variables. All of them obeyed American Society of Anesthesiologists fasting guideline. Intravenous access was secured with cannula in their wards.

After presenting to the operating room, the standard monitoring was utilized to pay close attention to vital signs, for example, heart rate (HR), pulse oxygen saturation (SpO_2_%), electrocardiogram (ECG) and noninvasive blood pressure (NIBP). These signs were monitored continuously and recorded automatically with 5min interval. Entropy index monitor was performed routinely before GA induction.

GA was induced with 0.03~0.05mg/kg midazolam, 2~2.5mg/kg propofol, 0.3~0.4ug/kg sulfentanil and relaxed with 0.6~1.0mg/kg rocuronium. At the same time, facial mask preoxygenation was applied with 100% O_2_ for 5min. If hypoventilation or loss of consciousness, ventilation assistance was initiated. After absolute muscle relaxation, endotracheal intubation was completed via a video-laryngoscope. Mechanical ventilation parameters were set with a tidal volume of 6~10ml/kg, 1:2 ratio of inspiration and expiration and respiratory rate of 10~20 per min to maintain end tidal pressure of CO_2_ 35~45mmHg. After anesthesia induction, radial arterial cannulation was implemented for continuous arterial pressure monitor. Meanwhile oesophagus temperature probe and warm dryer were used to keep patient body temperature between 36~37℃.

Anesthesia was maintained accordance with different anesthesia protocols. In the S group, patients were accepted sevoflurane, sulfentanil and rocuronium. And in the P group, propofol was instead for sevoflurane. All of these anesthetics were ceased on the end of operation. During the operation, the injection speed of anesthetics and concentration of sevoflurane was regulated to keep entropy index between 40~60 and the shift of mean arterial pressure (MAP) less than 20% of basic level. All patients were operated by the same surgical team with two decades experience of CP resection.

The primary outcome measures were the levels of hormones, including glucocorticoid (GC), ACTH, TSH, TT3, TT4, FT3 and FT4. These hormones were evaluated on preoperative time as basal level (T0), 1h after the beginning of operation (T1), the end of operation instantly (T2) and 72h after operation (T3). The secondary outcomes measures were duration of operation, total fluid infusion volume, urine volume, amount of bleeding, volumes of allogeneic and autologous transfusion. The MAP and HR on arrival in the operating room, the end of operation, and the peak values of them were included in the secondary outcome measures. At the same time, hospital stay and complications were also recorded, such as postoperative nausea, cerebral edema, and cerebral or lung infection.

**Safety consideration:**

All enrolled patient would undergo the standard general anesthesia as follow the previous. Consequently, there was no additional adverse event caused by the procedure of surgery and anesthesia. We keep the safety of participants in mind always, this is why we explore the best style of anesthesia in theses CP children. At the same time, we would follow up their postoperative complications.

**Follow up:**

In this study, all the enrolled patients would be followed up till postoperative 72h including their hormones levels and postoperative complications. Actually, all patients should be treated till discharge.

**Data management and statistics analysis:**

The study and its data were supervised by the Division of Science and Research of Sanbo Brain Hospital and the Ethic Committee. All data was collected via Electronic Record System. The statistics analysis of data was used with IBM SPSS Statistics V.21.0 (IBM Corp. Beijing, China). Continuous numerical variables were expressed as mean±standard deviation (SD), whose normality was assessed with Shapiro-Wilk test and histogram. Homogeneity of variance was evaluated by Levene’s test, and the means of continuous variables were compared via independent *t* test or Mann-Whitney *U* test where appropriate. Qualitative variables were presented as frequencies and percentages, and these variables were evaluated with Pearson *χ^2^* test or Fisher exact test. A *P* value less than 0.05 was considered statistically significant. Based on the pilot study, the sample size was 64 children which calculated with a power of 0.9, alpha error of 0.05 and 10% dropout rate.

**Quality assurance:**

The study was supervised by the Division of Science and Research of Sanbo Brian Hospital and the Ethic Committee.

**Expected outcomes of the study:**

The study would be published after completion. The more important is that the results are beneficial to select more suitable method or anesthetics of general anesthesia for CP children.

**Dissemination of results and publication policy:**

The results of this study would be published in specialty journal with Open Access.

**Duration of the project:**

The study was started after clinical trial registration and ethic approval from January to May 2022. The statistics analysis of data and paper writing were finished on next.

**Problems anticipated:**

Because this tertiary teaching hospital is very famous for CP resection in China, even worldwide, thus there is no difficult in this study. The aim of this study is to make better treatment.

**Project management:**

Jun Xiong and Mengrui Wang designed this clinical trial and wrote the primary article. Other anesthesiologists were responsible for group allocation but not involved in the further study. Yafen Zhou and Yanan Pang were responsible for statistics analysis, who were blind to the group allocation. Yongxing Sun took charge of funding support for the study and gave advices to English writing.

**Ethics:**

This trial was approved by the Ethics Committee of Sanbo Brain Hospital, Capital Medical University, SBNK-YJ-2021-030-01. Before recruiting participants, all guardians of children were informed the detailed content of this study and inquired whether took part in this study voluntarily. If they agreed, the informed written consent was signed.

**Informed consent forms:**

There is a copy of informed written consent form.
