# Supplementary material for: Propofol suppresses hormones levels more obviously than sevoflurane in pediatric patients with craniopharyngioma: A prospective randomized controlled clinical trial
Source: PLoS One. 2023 Jul 28;18(7):e0288863. doi: 10.1371/journal.pone.0288863 (PMC10381029; doi:10.1371/journal.pone.0288863)
Supplement: S2 File — (PDF) [file pone.0288863.s003.pdf]

Propofol suppresses hormones levels more obviously than sevoflurane in pediatric patients with craniopharyngioma: A prospective randomized controlled clinical trial

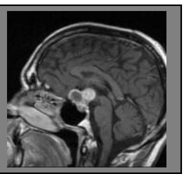

Methods and Material

Children with CP undergoing neurosurgery

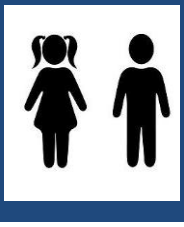

GA with sevoflurane and propofol respectively (n=32)

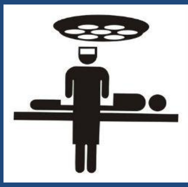

Evaluating hormones levels perioperatively

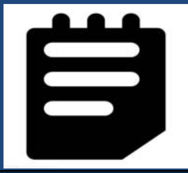

Findings

Hormones reduced by surgery and general anesthesia

Propofol affected hormones more obviously

Thyroid hormone and ACTH reduced significantly

Limitation

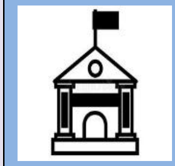

Single center trial

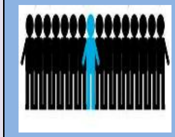

Small sample size

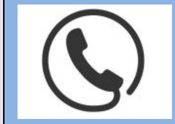

No long-term follow-up

Conclusion: Propofol and sevoflurane could reduce the levels of hormones intraoperativly and postoperatively in pediatric patients with craniopharyngioma, sevoflurane might be the preferred anesthetic because of less interruption on hormones levels
