# Supplementary material for: Propofol suppresses hormones levels more obviously than sevoflurane in pediatric patients with craniopharyngioma: A prospective randomized controlled clinical trial
Source: PLoS One. 2023 Jul 28;18(7):e0288863. doi: 10.1371/journal.pone.0288863 (PMC10381029; doi:10.1371/journal.pone.0288863)
Supplement: Attachment — Submitted filename: Response to Reviewers.doc [file pone.0288863.s007.doc]

**Response to reviewers**

Dear editor Jean Baptiste Lascarrou and reviewers:

We sincerely thank you and all reviewers for your valuable feedback again. Your comments and suggestions are very useful for improvement of our manuscript revision and future studies. Please do forward our heartfelt thanks to these experts.

Based on all comments we received, careful modifications have been made in the revision manuscript. All changes were marked with red text. In addition, we invited a native English speaker to check our English writing again. We tried our best to revise our manuscript to meet your standard. Below you will find our point-by-point responses to the academic editor and reviewers’ comments or questions:

Response to academic editor’ comments or questions:

1. Please review your reference list to ensure that it is complete and correct. If you have cited papers that have been retracted, please include the rationale for doing so in the manuscript text, or remove these references and replace them with relevant current references. Any changes to the reference list should be mentioned in the rebuttal letter that accompanies your revised manuscript. If you need to cite a retracted article, indicate the article’s retracted status in the References list and also include a citation and full reference for the retraction notice.

Response: Appreciate you for your academic reminder. We read all references in our manuscript and made sure they were complete and correct. And according with the reviewer’s comment, we also added references in the part of discussion. There was no retracted paper to be cited in our manuscript.

2. The manuscript has been improved, even some remarks remained. Please address reviewer's remarks and provide a graphical abstract if possible.

Response: Thanks for your comments. During the previous round of revision submission, we already provided a graphical abstract according on your suggestion.

Response to reviewers' comments and questions:

1. Is the manuscript presented in an intelligible fashion and written in standard English?

PLOS ONE does not copyedit accepted manuscripts, so the language in submitted articles must be clear, correct, and unambiguous. Any typographical or grammatical errors should be corrected at revision, so please note any specific errors here.

Reviewer #1: (No Response)

Reviewer #2: No

Response: Thank you for your comments. We were sorry for this problem because English is not our native language. We tried our best to revise our manuscript and invited a native English speaker to assist our previous round of revision. Although English writing is a difficulty, we have never stopped to improve. We paid more attention on English writing in this revision, and hope our revision to be close to standard English step by step.

2. Reviewer #1: If Mann-Whitney method is used, report median (range) in addition to mean (SD) and put a note on the table.

Response: Thanks for your suggestion. Non-normal data should be expressed as median (range) or median (quartile). Because these data of hormone levels were continuous numerical variables, so they were expressed as the mean±SD. And this expression style could keep table2 and table3 in the unified form. According with your constructive advice, we checked the primitive statistical data carefully and added the median (range) in addition to the mean (SD). Moreover, these changes were marked on the tables.

3. Reviewer #2: Thank you for the opportunity to review the revised manuscript by Drs. Xiong and colleagues. Please provide additional contextual evidence that more clearly and impact fully describes the clinical significance of decreased pituitary hormones intraoperatively, which is your main outcome finding. Is there any known impact on morbidity and mortality, and clinical outcomes? Is there any clinically meaningful effect of less reduced pituitary hormones in the preoperative period for craniopharyngioma surgery under sevoflurane anesthesia compared to propofol anesthesia? Please comment on this potential impact in the Discussion.

Response: We appreciate your constructive suggestion. Our study did demonstrate propofol and sevoflurane inhibiting pituitary hormones intraoperatively, but what consequences caused by this transient inhibition was litter known. We referred to and increased references related to hypopituitarism in our revised manuscript again. If diagnosis and treatment of hormones deficiency were delayed, clinical prognosis would be bad, even life-threating. Because hormone deficiency could be confirmed and hormone replacement could be immediately started postoperatively in our institution, thus long-term effects of anesthetics inhibiting pituitary hormones on morbidity and mortality of craniopharyngioma would need long-term follow-up. Although a lack of long-term follow-up is one limitation of this study, it is our work in the future study.

On behalf of all the contributing authors, I would like to express our sincere appreciations again. According to these valuable and constructive comments, we have made extensive modifications to our manuscript. If there any other revisions we could make, we would like very much to modify them. We hope the revised manuscript might meet your requirements, though there is a gap between them. Thus, we look forward to your warm comments again.

Yours sincerely

Correspondence author: Yongxing Sun

Email address: b2008194@126.com

Telephone number: 086-13810197854
